# Encouragement

**Published:** 1875-09

**Authors:** 


*Encouragement.*

"The August number of the RECORD has been received. Read with more than usual interest on account of its delay. We found it had shared the fate of many that had preceded it, but we are now satisfied that its editors are doing their duty, and as one of its readers from its commencement, I promise in future to pay the subscription fee in advance, and to do all in my power to increase its circulation. I will send you at least two new subscribers for next year. We hope the RECORD will continue to urge the importance of the ethics being observed and enforced in every State. The private means used by sending circulars to physicians and ministers to advertise medical colleges and to deceive the ignorant, has thoroughly disgusted the profession in this section, and we have determined to advise our students to attend institutions of established reputation—those that present their claims in a decent and professional manner, and through the regular and authorized channels. Members of organized colleges should not be allowed to use means to advance their interest that are not allowed to be used by private practitioners for the same purpose. If something is not done to give protection to those who have moral courage enough to sustain the ethics and place these medical demogogues and tracters where they justly belong, the good men will lose all respect for the ethics and cease to attend medical associations."

The above views and expressions of one of our co-laborers are too true to need comment. We publish them with the hope that it may influence the profession, as a "little leaven leaveneth the whole lump." P.

